# The effect of music to improve sleep quality in depression related insomnia

**DOI:** 10.1192/j.eurpsy.2023.514

**Published:** 2023-07-19

**Authors:** H. N. Lund, I. N. Pedersen, A. Heymann-Szlachcinska, M. Tuszewska, G. Bizik, J. I. Larsen, A. Drago, E. Kulhay, A. Larsen, H. Ø. Sørensen, B. Grønbech, L. R. Bertelsen, J. B. Valentin, J. Mainz, S. P. Johnsen, N. Hannibal, R. MacDonald

**Affiliations:** ^1^Psychiatry - Unit for depression, Aalborg University Hospital; ^2^Psychiatry, Aalborg University, Music Therapy Clinic; ^3^Psychiatry, Aalborg University Hospital; ^4^ Danish Center for Clinical Health Services Research, Aalborg University; ^5^Psychiatry Management, Aalborg University Hospital, Aalborg, Denmark; ^6^Reid School of Music, The University of Edinburgh, Edinburgh, United Kingdom

## Abstract

**Introduction:**

Insomnia in depression is common and difficult to resolve. Music is commonly used as a sleep aid, and clinical trials pointing to positive effects of music as a sleep aid are increasing adding to the evidence base. There is little knowledge on the effectiveness of music for depression related insomnia.

**Objectives:**

A recent RCT study conducted in psychiatry at Aalborg University Hospital examined effects of a music intervention for insomnia in depression. The intervention group listened to music at bedtime for four weeks, controls were offered music intervention post-test. Primary outcome measure was Pittsburgh Sleep Quality Index (PSQI). Secondary outcomes included Actigraphy, The Hamilton depression Rating Scale (HAMD-17) and World Health Organisation well-being questionnaires (WHO-5, WHOQOL-BREF).

**Methods:**

A two-armed randomized controlled trial (n=112) and a qualitative interview study (n=4)

**Results:**

The RCT study showed signficant improvements for the music intervention group in sleep quality and quality of life at four weeks according to global PSQI scores (effect size= -2.1, 95%CI -3.3; -0.9) and WHO-5 scores (effect size 8.4, 95%CI 2.7; 14.0). Actigraphy measures showed no changes and changes in depression symptoms (HAMD-17) were not detected.

The interview study unfolded examples of the influences of music on sleep and relaxation. Music distracted, affected mood and arousal positively and supported formation of sleep habits.

Results from the trial are discussed and merged with findings from the interview study. The results from the trial suggested moderate effects of music listening for the population while findings from the interview study showed examples of individual and highly varying outcomes.

**Conclusions:**

Music is suggested as a low-cost, side-effect free and safe intervention in supplement to existing treatments improving sleep in depression.

**Disclosure of Interest:**

None Declared

